# Two Case Reports of Scombroid in Singapore: A Literature Review

**DOI:** 10.7759/cureus.22580

**Published:** 2022-02-24

**Authors:** Roshan M Lalmalani, Jaryl Gan HS, Simon Stacey

**Affiliations:** 1 General Medicine/Geriatric Medicine, Sengkang General Hospital, Singapore, SGP; 2 General Medicine, Sengkang General Hospital, Singapore, SGP

**Keywords:** foodborne diseases, histamine fish poisoning, fish allergy, scombrotoxin, scombroid

## Abstract

Scombroid is a foodborne illness that results from eating improperly handled fish. Due to a disruption in the cold chain, these fish have high histamine levels. As a result, scombroid presents with allergy-like symptoms but is not really an allergy per se. Cases have been reported in many countries.

Here, we report two cases of a 48 and 17-year-old father and son in Singapore who developed symptoms suggestive of scombroid after eating tuna imported from Vietnam delivered by an internationally known supply company. The diagnosis was confirmed by elevated histamine levels measured in the culprit fish product. We discuss the pathophysiology, signs, symptoms, and management of scombroid.

## Introduction

Scombrotoxin fish poisoning (SFP) also known as scombroid poisoning, scombrotoxicosis, or histamine fish poisoning is a foodborne illness that results from the consumption of fish that has been improperly handled between the time it is caught and the time it is cooked [1]. The word “scombroid” is derived from Scombridae which is a family of dark-fleshed fish consisting of species such as mackerel and tuna. However, non-scombroid fishes such as mahi-mahi, salmon, and sardine have also been implicated in scombroid poisoning [2].

Scombroid poisoning is very common. A 2013 report from the United States estimated over 35,000 cases resulting in 162 hospital admissions between 2000 and 2009 [3]. Scombroid cases have also been reported from countries such as Australia [4], the Netherlands [5], Israel [6], Colombia [7], and many others.

Inappropriate storage, resulting in disruption of the cold chain, of the fish leads to bacterial enzymatic conversion of free histidine into histamine. This is due to the action of bacterial histamine decarboxylase (HDC), usually by mesophilic bacteria such as *Clostridium perfringens*, *Morganella morganii*, etc. As a result, high levels of histamine are usually found in the culprit seafood item [8]. Whether histamine is the only constituent of “scombrotoxin” is unclear. Nevertheless, the symptomatology is essentially that of histamine toxicity. It is considered an atypical foodborne illness as the main symptoms are not gastrointestinal and also because it is not due to contamination of the product.

While mostly self-limiting and mild, there have been reports of life-threatening scombroid poisoning. A previously healthy young woman developed hypotension needing vasopressors with ST depressions [9] while another scombroid poisoning was complicated by acute pancreatitis [10]. Some cases were severe enough to need ICU admissions. A recent narrative discussed acute coronary syndromes (ACS) associated with scombroid. Of note, there is a potential of hemodynamic failure in the acute stage, even in apparently healthy people [11].

Closer to home, in September 2016, the Agri-Food and Veterinary Authority (AVA) of Singapore issued a recall order on a batch of canned tuna imported from Thailand under a common food brand. This was reported in various newspapers such as The Straits Times and The Independent. In one issue of the Singapore Food Agency’s Food Safety Bulletin in 2018, there was a segment on scombroid. Interestingly, however, a PubMed search was conducted and while we found a case of pufferfish poisoning reported in 2013 [12] and a report of stonefish poisoning in 2009 [13], we found no reports of scombroid poisoning in Singapore. We report two confirmed cases of scombroid poisoning who are from the same household who presented to our hospital after consuming tuna for dinner.

## Case presentation

Two patients were admitted from the emergency department of our hospital for symptoms occurring shortly after a meal consisting of tuna. They are father and son and ended up being admitted into the same ward.

Case 1

A 48-year-old man, with a past medical history of hypertension, hyperlipidemia, diabetes, and psoriasis presented with sudden onset of a generalized itchy rash, dizziness with a near syncopal episode, and swelling of his lips. The symptoms started within one hour after his dinner which consisted of tuna. When he felt dizzy, his home blood pressure machine showed a reading of 80/60 mmHg.

His blood pressure on arrival at the emergency department was 96/48 mmHg with a heart rate of 113 beats/minute. He was not in respiratory distress. There was no wheeze on auscultation. There was a generalized maculopapular rash over his abdomen and all four limbs. There was no periorbital, lip, or uvula swelling. His ECG showed sinus tachycardia. Aside from a very slightly raised total white blood cells of 10.13 thousand cells per cubic millimeter of blood with a normal eosinophil count of 0.41 thousand cells per cubic millimeter of blood, his full blood count, renal function test, and chest radiograph were unremarkable. He was given two doses of intramuscular adrenaline (0.3 mg each), 100 mg of intravenous hydrocortisone, 20 mg of intravenous famotidine, and given fluid resuscitation in the form of 500 ml each of normal saline and compounded sodium lactate infusions.

During observation in the emergency department, he complained of chest discomfort. His lungs remained clear and a nasopharyngolaryngoscopy revealed no signs of upper airway obstruction. He was then admitted to the inpatient ward. His stay remained uneventful and he was discharged the next morning with a five-day course of oral prednisolone, fexofenadine, and famotidine. 

Case 2

A 17-year-old man, who has a known allergy to peanuts, with no past medical history presented with sudden onset of generalized pruritic rash, upper abdominal pain, nausea, and headache. The symptoms started around 30 minutes after dinner which consisted of the same tuna as case 1. His initial blood pressure was 126/80 mmHg, with a heart rate of 97 beats/minute. His oxygen saturation was 100% on room air. He was alert and comfortable. There was facial flushing and a generalized erythematous rash over his torso and limbs.

He had mild epigastric and right hypochondriac tenderness. There was guarding. His heart and lung examinations were unremarkable. He was neurologically intact. His full blood count, kidney and liver function tests were within normal limits. He was given 0.3 mg adrenaline intramuscularly along with hydrocortisone (100 mg), diphenhydramine (25 mg), and famotidine (20 mg) intravenously. He was then admitted to the ward where his stay was uneventful and he was then discharged the next morning. Having been seen by the same inpatient team, he too received a five-day course of oral prednisolone, fexofenadine, and famotidine.

Both the patients were discharged without a follow-up appointment. Of note, two other members in the family also consumed the same product but comparatively in very small amounts. They remained asymptomatic. Table 1 provides a summary of the cases.

**Table 1:** Comparative summaries of the cases. HR: heart rate; RR: respiratory rate; BP: blood pressure

	Case 1	Case 2
Age/gender	48 years/male	17 years/male
Past medical history	Hypertension, hyperlipidemia, type 2 diabetes mellitus, and psoriasis on monthly ixekizumab and topicals	Nil
Allergy history	No known drug or food allergy	Allergic to peanuts
Presenting complaints	Generalized itchy rash, lip swelling, and near syncope	Generalized rash, upper abdominal pain, nausea, and headache
Initial vitals	Afebrile, BP 96/48 mmHg, HR 113 beats/minute, RR 19 breaths/minute	Afebrile, BP 126/80 mmHg, HR 96 beats/minute, RR 23 breaths/minute
Treatment	2 doses of intramuscular adrenaline (total 0.6 mg), 100 mg intravenous hydrocortisone, 20 mg intravenous famotidine, 1 liter of intravenous fluid resuscitation, and admission to a general medical ward	1 dose of intramuscular adrenaline 0.3 mg, 100 mg intravenous hydrocortisone, 20 mg intravenous famotidine, 25 mg intravenous diphenhydramine, and admission to a general medical ward
Follow-up	Discharged after overnight stay with a 5-day course of oral steroids and antihistamines	Discharged after overnight stay with a 5-day course of oral steroids and antihistamines

The patients were instructed to keep a sample of the implicated seafood in their house. The cases were reported to the Singapore Food Agency (SFA) who subsequently collected samples from both the importer and the patients. These samples were sent to National Centre for Food Science (NCFS) to test for histamine. The sample from the patient revealed very high levels of histamine of 6556.68 parts per million (ppm). The SFA’s limit is 100 ppm. The samples from the distributor were within normal limits. Therefore, the cold chain was likely to have been breached at the consumer (patient)’s end. They were promptly advised to dispose off any frozen tuna they had left.

## Discussion

Scombroid is an under-reported foodborne illness caused by consuming improperly handled fish such as tuna and mackerel which belong to the Scombridae family or even non-scombroid fish such as salmon, mahi-mahi, or sardine. This results in a bacterial enzymatic production of high levels of histamine which results in a clinical presentation that is similar to an allergic reaction [14]. Symptoms usually start between 10 minutes to one hour after ingestion and include flushing, rash, dizziness, headache, conjunctival injection, palpitations, abdominal pain, nausea, vomiting, and diarrhea. Symptoms are usually self-limiting and rarely last beyond 24 hours [15]. Rarely, serious cases have been described. Treatment is supportive and antihistamines are usually administered. Adrenaline may have a role in the initial treatment, especially in the setting of hemodynamic compromise. Corticosteroids are usually not indicated [15,16]. However, in the initial presentation, differentiating serious scombroid from anaphylaxis is extremely difficult and corticosteroids may still need to be administered. In case 2, in view of hemodynamic stability, corticosteroids and adrenaline may not have been necessary.

Both of them presented with signs and symptoms which could have easily been attributed to allergy [14]. That would have resulted in both of them being wrongly labeled as having a seafood allergy which would have long-term implications. Table 2 attempts to differentiate scombroid and allergic reaction. A correct diagnosis is also important from a public health point of view as it can potentially prevent an outbreak if the mishandling of the product occurs at the supplier or even higher level. Therefore, people presenting to the emergency department with allergy-like symptoms should be questioned about the consumption of fish in the hours before onset of symptoms. If there are any suggestions of inappropriate storage or handling of the fish, it should then be sent for testing at a reference center. Many hospital labs do not routinely test this. 

**Table 2:** Differences between scombroid and allergic reaction.

Characteristic	Scombroid	Allergy
Patient population	Everybody who ate the contaminated product.	Usually one patient at a time.
Pathophysiology	Histamine-mediated.	Most commonly IgE-mediated.
Onset	10 minutes to 1 hour after ingestion.	Within a few minutes, although there are some late-phase allergic reactions which may even present few hours after exposure
Course	Usually mild and self-limiting; rare cases of severe reactions.	Potentially fatal.
Symptoms	Rash, pruritus, flushing, headache, abdominal pain, nausea, vomiting, diarrhea, dizziness, headache; hypotension and angioedema are uncommon.	Rash, pruritus, flushing, angioedema, nausea, vomiting abdominal pain, diarrhea, shortness of breath, stridor, hoarseness of voice, hypotension, shock.
Diagnosis	History of other people with similar reactions after eating the same food products, serum histamine levels in the affected seafood.	Anaphylaxis markers such as serum tryptase, skin testing.
Treatment	Supportive, antihistamines; adrenaline and corticosteroids not routinely indicated.	Adrenaline, supplemental oxygen, and fluid resuscitation as needed; adjuncts include salbutamol for bronchospasm, antihistamines, and corticosteroids.
Prevention	Correct handling of seafood products. Maintenance of cold chain.	Avoidance of culprit allergen.

In Singapore, SFA should be contacted for possible scombroid cases. In this case, neither the hospital nor the patients were charged for the test. Scombroid poisoning can be prevented by keeping fish refrigerated, thereby maintaining the cold chain [17]. The toxins that cause scombroid poisoning are heat-stable [18]. Thus, cooking of contaminated fish will not reduce the risk of illness.

## Conclusions

Scombroid is a foodborne illness that results from the consumption of fish with high levels of histamine. This usually occurs due to disruption in the cold chain. Symptoms of scombroid overlap with that of an allergic reaction and awareness of this condition coupled with a good history will help clinicians diagnose such cases. Confirmation is by testing the culprit food product at an official lab for histamine levels. Scombroid is generally self-limiting and treatment is supportive.
